# Screening of an anti-inflammatory peptide from *Hydrophis cyanocinctus* and analysis of its activities and mechanism in DSS-induced acute colitis

**DOI:** 10.1038/srep25672

**Published:** 2016-05-09

**Authors:** Zengjie Zheng, Hailong Jiang, Yan Huang, Jie Wang, Lei Qiu, Zhenlin Hu, Xingyuan Ma, Yiming Lu

**Affiliations:** 1 State Key Laboratory of Bioreactor Engineering, East China University of Science and Technology, Shanghai 200237, China; 2 Department of Biochemical Pharmacy, School of Pharmacy, Second Military Medical University, Shanghai 200433, China

## Abstract

Snake has been used for centuries as a traditional Chinese medicine, especially for therapeutic treatment for inflammatory diseases; however, its mechanisms of action and active constituents remain controversial. In our study, a tumor necrosis factor receptor 1 (TNFR1) selective binding peptide, Hydrostatin-SN1 (H-SN1), which was screened from a *Hydrophis cyanocinctus* venom gland T7 phage display library, was shown to exhibit significant anti-inflammatory activity *in vitro* and *in vivo*. As a TNFR1 antagonist, it reduced cytotoxicity mediated by TNF-α in L929 fibroblasts and effectively inhibited the combination between TNF-α with TNFR1 in surface plasmon resonance analysis. H-SN1 was also shown to suppress TNFR1–associated signaling pathways as it minimized TNF-α-induced NF-кB and MAPK activation in HEK293 embryonic kidney and HT29 adenocarcinoma cell lines. We next determined the effect of H-SN1 *in vivo* using a murine model of acute colitis induced by dextran sodium sulfate, demonstrating that H-SN1 lowered the clinical parameters of acute colitis including the disease activity index and histologic scores. H-SN1 also inhibited TNF/TNFR1 downstream targets at both mRNA and protein levels. These results indicate that H-SN1 might represent a suitable candidate for use in the treatment of TNF-α-associated inflammatory diseases such as inflammatory bowel diseases.

Snake has been used for centuries as a traditional Chinese medicine; however, its active constituents and mechanisms remain largely unknown. Research has demonstrated that snake venom is a mixture of proteins and peptides with various different biological activities^1^. In particular, sea snakes possess more streamlined and stable venom than land snakes^2^, thus providing an optimized resource for the identification of distinct bioactive components conducive to subsequent functional characterization and development.

Tumor necrosis factor (TNF-α, or TNF) is a highly pleiotropic cytokine that is involved in various autoimmune and inflammatory diseases such as inflammatory bowel disease, rheumatoid arthritis and septic shock^3^. The biological functions of TNF-α are mediated by two functionally distinct but structurally related cell membrane receptors, TNFR1 and TNFR2. TNFR1 is the primary signaling receptor on most cell types and associated with the majority of the cytotoxic, proinflammatory, and apoptotic effects when activated by TNF-α^4^,^5^,^6^. In contrast, TNFR2 is reported to function as a critical role in sustaining Foxp3 expression and maintaining the phenotypic and functional stability of the regulatory T cell pool that is required for immune regulation and to avoid harmful damage to self-tissues^7^,^8^,^9^. Therefore, in our study, we selected TNFR1 as the specific target for screening bioactive peptides from *Hydrophis cyanocinct*us venom gland T7 phage display library.

Inflammatory bowel diseases (IBDs) comprise a series of chronic inflammatory diseases that seriously affect the gastrointestinal tract; these have been mainly subdivided as Crohn’s disease and ulcerative colitis. Patients suffering from IBD undergo a group of clinical symptoms including diarrhea, rectal bleeding, and weight loss^10^, and their life is also affected by complications such as bowel perforation^11^, toxic megacolon^12^, and surgical complications^13^. Furthermore, IBDs often cause a long-term even irreversible impairment in intestinal structure and function. Notably, various lines of evidence have demonstrated a close inherent connection between colitis and colon neoplasms. Although the etiology is not completely clear, IBDs are usually deemed to be induced by uncontrolled inflammatory processes in the intestines.

As a pleiotropic cytokine, the upregulation of TNF-α expression is a hallmark of inflammatory response. Accordingly, although the etiology of Crohn’s disease remains unclear, a series of research have indicated that TNF-α functions as a core role in the inflammatory response in this disease. Notably, the level of TNF-α is increased in the stool and serum of patients suffering from Crohn’s disease^11^,^12^. As substantial evidence indicates that TNFR1 signaling is pathologic whereas TNFR2-dependent signaling might play an anti-inflammatory role, treatment specificity might be achieved through inhibiting the transmembrane TNF/TNFR1 signaling while leaving the often beneficial TNF/TNFR2 axis mechanism unaffected. This approach appears promising as it would be expected to promote inhibition of the pathological effects of TNF-α while leaving the TNF/TNFR2 axis active, while further raising the possibility for the treatment of other diseases in which TNFR2 inhibition is detrimental.

Therefore, to identify likely compounds that would selectively inhibit TNFR1, in our study we selected TNFR1 as the specific target for screening bioactive peptides from a *Hydrophis cyanocinctus* venom gland T7 phage display library. To examine the potential anti-inflammatory actions of the screened compounds, the effects of the TNFR1-binding peptide H-SN1 were then determined using the dextran sulfate sodium (DSS) induced colitis murine model. Several correlations between the model and human IBDs have been identified; thus, this model has been considered suitable for investigating the pathogenesis and therapeutic options for IBDs^14^. Our results showed that H-SN1 was able to significantly ameliorate the correlative symptoms of colitis in the mice, effectively alleviate the colonic pathological damage, and also to affect the downstream targets of the TNF/TNFR1 axis at both the gene and protein levels.

## Results

### *H. cyanocinctus* venom gland T7 phage display library construction and biopanning

The construction of *H. cyanocinctus* venom gland T7 phage display library was following the guidelines of the OrientExpress cDNA and T7Select System Manuals (Novagen, Madison, WI, USA) as shown in Materials and Methods. The titer of the original library was 1.56 × 10^6^ pfu/ml.

One potential binding peptide was selected after sequencing, named Hydrostatin-SN1, with the nucleotide sequence: 5′-GAC GAA CAA CAC CTA GAG ACC GAA CTA CAC ACT CTC ACC AGC GTG CTG ACA GCC AAT GGA TTC CAA-3′ corresponding to the amino acid sequence: DEQHLETELHTHLTSVLTANGFQ.

### Structure of H-SN1 and the docking model

**Figure 1:**
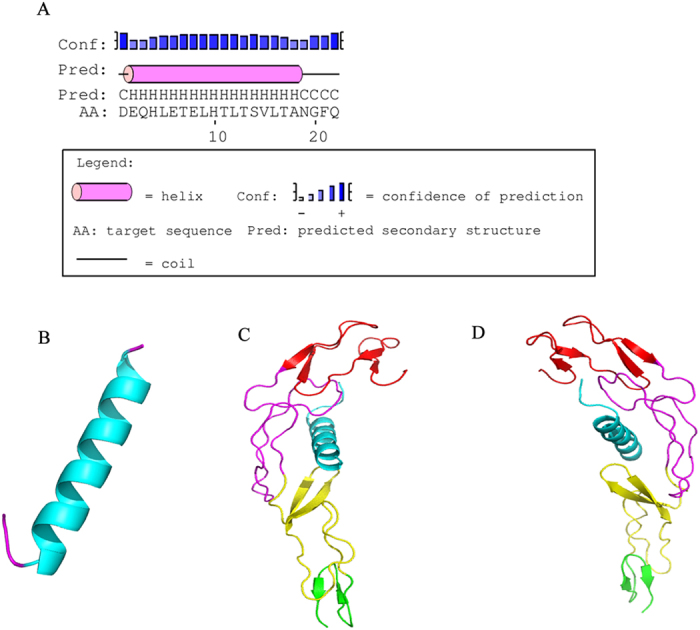
Structure prediction and docking model. (**A**) Secondary structure of H-SN1. (**B**) Three dimensional structure of H-SN1. Molecular docking structure of H-SN1 with TNFR1 viewed from the left (**C**) and the right (**D**). The CRD1, CRD2, CRD3, and CRD4 domains are shown in red, magenta, yellow, and green, respectively. H-SN1 is shown in cyan.

As shown in Fig. 1A, the main region of H-SN1 was assumed to adopt an α-helical conformation. The *de-novo* prediction of the three dimensional structure of H-SN1 showed a coil-helix-coil conformation (Fig. 1B), which was consistent with that in Fig. 1A. According to the molecular docking analysis, H-SN1 bound the cysteine-rich domains (CRDs) 2 and 3 in TNFR1, which represent the binding regions of TNF-α (Fig. 1C,D).

### Binding properties of H-SN1

**Figure 2:**
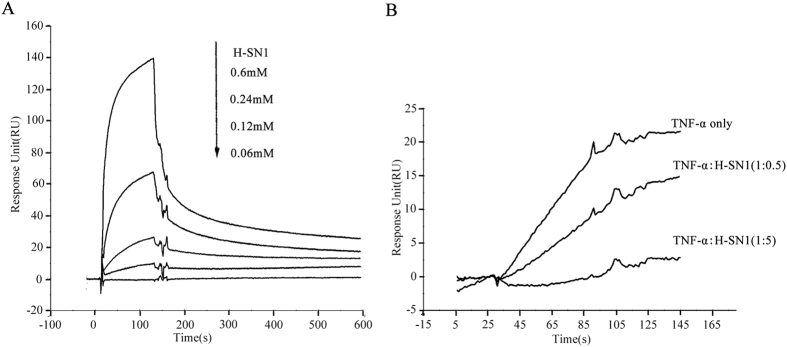
H-SN1 exhibits TNFR1 binding capability and inhibits TNF-α/TNFR1 interaction. (**A**) BIAcore assay for H-SN1 binding to TNFR1 coupled to a CM5 biosensor chip. (**B**) The response change of TNF-α binding to immobilized TNFR1 was measured in the presence of increasing concentrations of H-SN1.

We investigated whether H-SN1 targets TNF-α or inhibits TNF-α binding to TNFR1 using surface plasmon resonance (SPR) analysis. In our study, BIAcore T100 and CM5 sensor chips were utilized. H-SN1 resulted in a dose-dependent resonance when flowed through TNFR1 immobilized on a biosensor chip, demonstrating the direct combination of H-SN1 with TNFR1 (Fig. 2A). BIAevaluation 3.2 software was used to analyze the binding curves, and K_D_ was determined to be 32 μM. Kinetic measurement results indicated that H-SN1 did not exhibit a strong affinity to TNFR2 (data not shown). Further competition assays showed that H-SN1 exhibited a marked influence on TNFR1-blockage in a dose-dependent manner (Fig. 2B).

### Effect of H-SN1 on TNF-α-induced cytotoxicity in L929 fibroblasts

**Figure 3:**
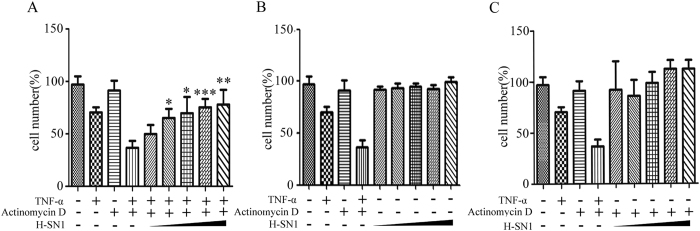
H-SN1 neutralizes TNF-α-induced cytotoxicity in L929 cells without exhibiting independent cytotoxic effects. Actinomycin D-treated L929 cells plus TNF-α were incubated with various concentrations of H-SN1 (32 pM, or 0.16, 0.8, 4, or 2 nM) for 18–20 h and stained with MTT (**A**). Absorbance was determined at 490 nm. The cytotoxic effect of H-SN1 alone in L929 cells was determined by MTT assays without (**B**) or with (**C**) actinomycin D. Values represent the means ± SEM. *p < 0.05, **p < 0.01, ***p < 0.001 for the cells treated with H-SN1 vs. the cells treated with TNF-α + actinomycin D.

We evaluated the effect of H-SN1 on the biological activity of TNF-α using a TNF-α-mediated L929 cytotoxicity assay^15^,^16^. L929 cells are sensitized by actinomycin D. The treatment of TNF-α with actinomycin D resulted in almost 70% cytotoxicity in L929 cell, whereas treatment with actinomycin D alone did not show a significant cell killing effect (Fig. 3A). H-SN1 was able to inhibit TNF-α-induced cytotoxicity of L929 cells concentration-dependently. To test the cytotoxicity of H-SN1 further, the same process was performed with an MTT assay, which demonstrated that H-SN1 was not associated with significant cytotoxicity in L929 cells either with (Fig. 3C) or without (Fig. 3B) actinomycin D.

### Effect of H-SN1 on TNF-α-mediated activation of NF-κB and MAPK pathways

**Figure 4:**
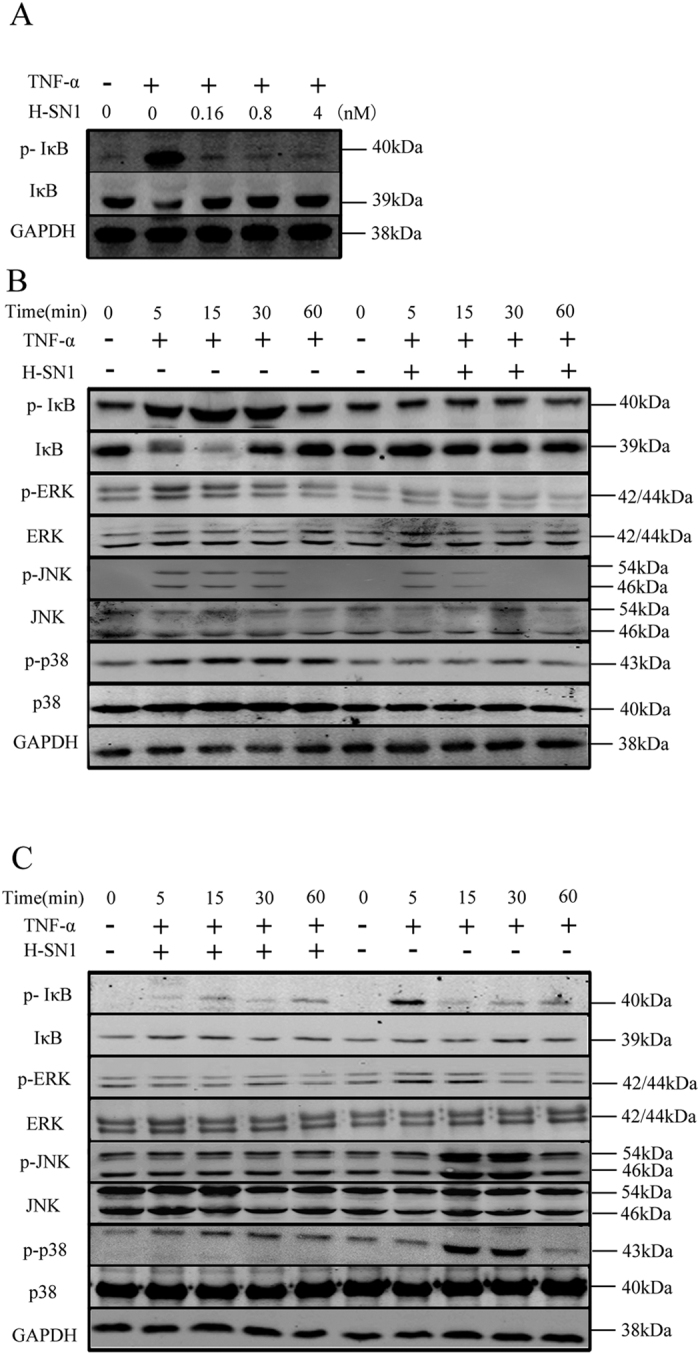
H-SN1 inhibits TNF-α-induced NF-κB and MAPK signaling activation. (**A**) HT-29 cells were treated with 20 ng/ml TNF-α for 10 min in the presence of various concentrations of H-SN1 (0.16, 0.8, and 4 nM). TNF-α was added to HEK293 cells (**B**) and HT-29 cells (**C**) in the presence or absence of 4 nM H-SN1 for different times. Original blots are shown in Supplementary Fig. S1; the gels were run under the same experimental conditions.

NF-кB and MAPK signaling triggered by the TNF-α/TNFR1 combination is associated with a series of proinflammatory functions of TNF-α^17^. We therefore determined whether H-SN1 could inhibit the proinflammatory effects of TNF-α in the HEK293 and HT29 cell lines by assessing these two signaling transduction pathways. Western blot results showed that TNF-α significantly increased the phosphorylation and degradation of IκB, which is an inhibitor of NF-кB. Treatment with H-SN1 significantly inhibited these changes dose-dependently (Fig. 4A, Supplementary Fig. S1A).

To evaluate the inhibitory chronergy of H-SN1 on TNF-α-mediated NF-κB and MAPK pathway stimulation, 20 ng/ml TNF-α was added to the HEK293 and HT29 cells in the presence of 4 nM H-SN1. As shown in Fig. 4B,C (Supplementary Fig. S1.B, C), the phosphorylation of IκB, p38, ERK and JNK in HEK293 and HT29 cells was significantly induced by TNF-α. This phenomenon was prevented by treatment with H-SN1 time-dependently.

### H-SN1 reduces inflammation in a DSS-induced murine colitis model

The DSS-induced murine colitis model was chosen for the assessment of the anti-inflammatory effects of H-SN1 *in vivo*. Compared with mice treated with DSS alone, mice with administration of H-SN1 presented a significant improvement.

**Figure 5:**
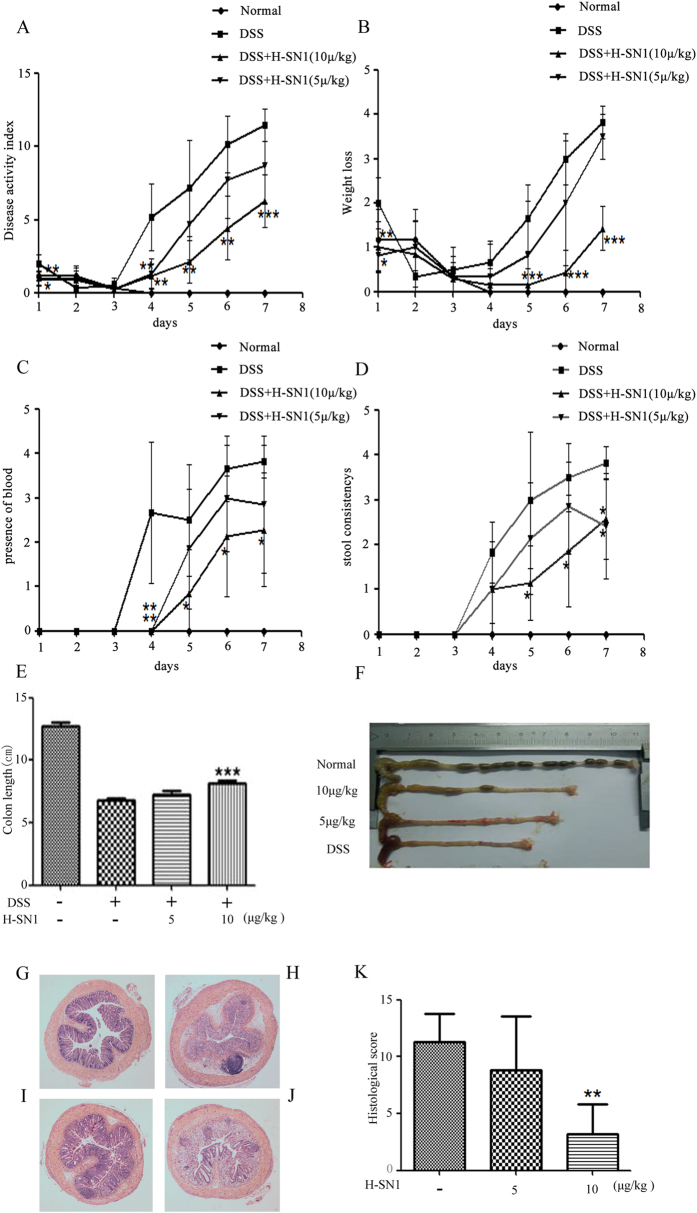
H-SN1 improves clinical parameters and reduces histologic injury in DSS-induced acute colitis. (**A**) BALB/c mice received DSS for 7 days, and DAI (as shown in Table 1) was monitored. Effects of daily intraperitoneal injections of H-SN1 on weight loss (**B**), fecal bleeding (**C**) and recovery from diarrhea (**D**). (**E**,**F**) Effect of H-SN1 on colon length in mice with DSS-induced acute colitis as assessed by histopathological changes: (**G**) Normal control mice; (**H**) DSS treated mice; (**I**) DSS + 10 μg/kg H-SN1 treated mice; and (**J**) DSS + 5 μg/kg H-SN1 treated mice. (**K**) Total histologic score. Values represent the means ± SEM, *p < 0.05, **p < 0.01, ***p < 0.001 for DSS + H-SN1 compared with DSS + vehicle. Hematoxylin & eosin staining magnification 40×.

During the entire experiment, the disease activity index (DAI) was monitored each day. As shown in Fig. 5A, DSS administration resulted in significant increases in diarrhea, rectal bleeding, and weight loss, whereas in mice treated intraperitoneally once daily with 10 μg/kg H-SN1, weight loss and diarrhea were significantly reduced by day 5~7 (Fig. 5B,D), and rectal bleeding was significantly reduced by day 4~7 (Fig. 5C). Furthermore, we found that colon length, a macroscopic indicator of colitis severity, in DSS-treated mice was significantly reduced compared with the control group (Fig. 5E,F). In contrast, administration of H-SN1 at 10 μg/kg led to a marked improvement in colon length (*p* < 0.001), but a low concentration of H-SN1 (5 μg/kg) had little effect on colon length reduction.

Histological injury was also ameliorated by treatment with H-SN1. As shown in Fig. 5G,H, compared with the normal group, DSS-induced acute colitis exhibited severely inflammatory cellular infiltration, submucosal edema and large areas of complete destruction of the epithelial structure including loss of crypts and epithelial integrity under microscopic examination. Administration of 10 μg/kg H-SN1 reduced the epithelial destruction, recovered the structure of the crypts, and reduced the degree of inflammation. After treatment with DSS the histological colitis scores increased to 11.3, whereas the administration of H-SN1 at 5 and 10 μg/kg reduced the scores to 8.83 and 3.16, respectively (*p < 0.01 at 10 μg/kg).

**Figure 6:**
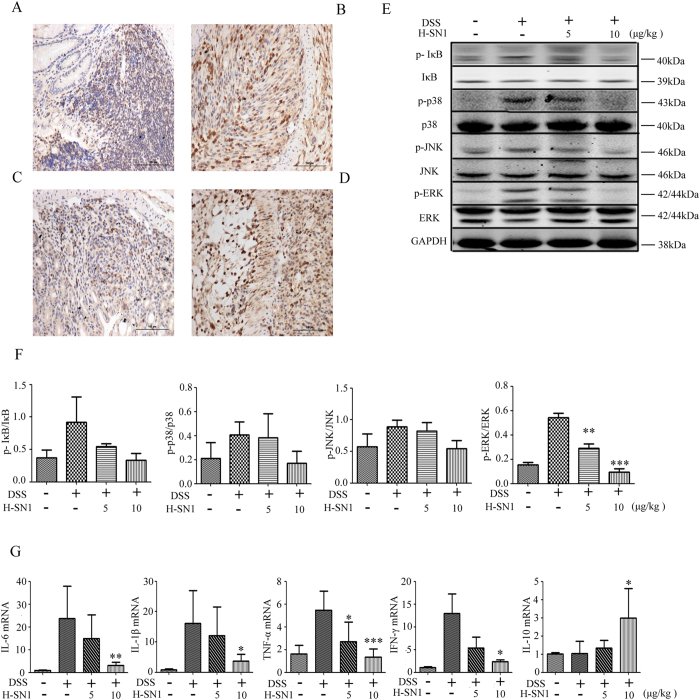
H-SN1 reduces TNF/TNFR1 axis downstream targets at both the gene and protein levels. H-SN1 effect on TNF-α expression in the colonic tissue in (**A**) Normal; (**B**) DSS; and (**C,D**) DSS + H-SN1, 10 and 5 μg/kg groups, respectively. H-SN1 effect on TNF-α induced the activation of NF-κB and MAPK in the colon of mice suffering from colitis. (**E**) Western blot analysis. (**F**) ImageJ software was used to quantify the levels of protein phosphorylation. (**G**) Impact of H-SN1 on the mRNA expression of inflammatory factors including IL-6, IL-1β, TNF-α, INFγ, and IL-10. Values represent the means ± SEM,*p < 0.05, **p < 0.01, ***p < 0.001 for DSS + H-SN1 compared with DSS + vehicle. Original blots are shown in Supplementary Fig. S2; the gels were run under the same experimental conditions.

TNF-α, as an important proinflammatory mediator, exerts a vital function in colitis. The secretion of TNF-α in turn improves the expression of the cytokine via positive feedback. Therefore, we tested whether the blockage of TNFR1 affects TNF-α expression by disturbing the positive feedback loop. Accordingly, TNF-α expression was significantly raised in the DSS-group (Fig. 6B), whereas administration of 10 μg/kg H-SN1 markedly reduced TNF-α expression in colonic epithelium tissue (Fig. 6C), which indicated the anti-inflammatory effect of H-SN1. In contrast, administration of 5 μg/kg H-SN1 had little impact on TNF-α expression (Fig. 6D).

To understand the mechanism of H-SN1 function *in vivo*, we investigated whether treatment with H-SN1 inhibited the NF-κB and MAPK pathways in the colonic tissue of mice suffering from colitis. As shown in Fig. 6E, (Supplementary Fig. S2) the phosphorylation of IκB, ERK1/2, p38 and JNK was significantly raised in the DSS group, whereas administration of 10 μg/kg H-SN1 was able to significantly reduce these changes (Fig. 6E,F, Supplementary Fig. S2). This result suggested that H-SN1 ameliorated DSS-induced acute colitis by inhibiting TNF-α-mediated activation of the NF-κB and MAPK pathways in the colons of mice.

Colonic inflammation consequent to the abnormal activation of immune cells is thought to lead to exorbitant secretion of proinflammatory cytokines^18^. In particular, the immune response has been believed to be aggravated with the binding of proinflammatory cytokines to immune cells in the intestine. Therefore, to gain further insight into the molecular mechanisms underlying the inhibition of colitis by H-SN1, the mRNA level of proinflammatory cytokines had been investigated. We found that DSS administration led to abnormal induction of IL-1β, TNF-α, IFN-γ, and IL-6 and that administration of 10 μg/kg H-SN1 significantly reduced these increases, whereas 5 μg/kg H-SN1 was not able to significantly prevent these increases (Fig. 6G). Furthermore, we quantified the mRNA expression of IL-10, an anti-inflammatory cytokine, from colon tissues. As shown in Fig. 6G, the expression of IL10 mRNA was not markedly induced by DSS whereas in the treatment group, 10 μg/kg H-SN1 significantly increased the mRNA expression of IL-10. Consistent with previous results, treatment with 5 μg/kg H-SN1 was not able to significantly induce this increase.

## Discussion

Through screening a *H. cyanocinctus* venom gland T7 phage display library, we discovered that H-SN1, a natural peptide, had inhibitory effect on TNF-α induced cytotoxicity in L929 cells dose-dependently, with no significant independent cytotoxicity on these cells with or without actinomycin D. In SPR analysis, we further indicated that H-SN1 could bind to TNFR1 at a K_D_ of 32 μM and markedly suppressed the combination of TNF-α with TNFR1 dose-dependently. These results indicated that H-SN1 could directly bind to TNFR1 and disrupt the combination between TNF-α with its receptor, thereby suppressing its activity. From the data in the article, the H-SN1 has been demonstrated to be a lead peptide and it is potential for the development of new agents for inhibitors of TNFR1 based on the peptide.

Increasing attention has been focused on the selective inhibition of TNF/TNFR1 axis, which is an alternative to complete blockage of TNF-α causing both TNFRs inhibition^19^. Receptor-selective but not complete blockage of TNF-α response is a therapeutic shift in the present clinical practice for inflammatory disease treatment. Conceptually, the selective inhibition strategy targets the predominant pathogenic pathway while leaving the signals for tissue homeostasis and immunocompetence unaffected. Over the long term, this would likely ameliorate therapeutic effect by reducing clinically undesired side effects such as the recurrence of tuberculosis and the risk of malignancies or increased rates in multiple sclerosis^20^, which occur probably owing to the lack of TNFR2-mediated myelin regeneration^9^. Our results demonstrated that H-SN1 effectively inhibited TNF/TNFR1 combination in SPR analysis and decreased TNF-α-induced NF-кB and MAPK activation in HEK293 and HT29 cells dose- and time-dependently.

To evaluate the anti-inflammatory activity of H-SN1 *in vivo*, the protective effect of H-SN1 in DSS-induced acute colitis in mice was assessed. TNF-α is well known to play a pivotal role in inflammatory response both in experimental colitis and in human IBDs. TNFR1 is an important signaling receptor that mediates the pro-inflammatory function of TNF-α^21^; accordingly, blockage of the biological function of TNF-α by TNF-α or TNFR1-binding peptides was shown to exhibit significant effect in TNBS-induced colitis^22^, and several clinical studies have also shown that an anti-TNF-α antibody exhibited high effectiveness and safety in the treatment of IBD^23^,^24^,^25^. In comparison, our data showed that treatment with H-SN1 at the attack of colitis significantly alleviated the severity of the wasting disease in clinical and pathological level, relieving body weight loss, bloody diarrhea, and serious intestinal inflammation. Furthermore, measurement of protein changes in the inflamed colon by western blotting showed that treatment with H-SN1 was able to inhibit the TNF-α-mediated NF-κB and MAPK proinflammatory pathway activation.

Following treatment with H-SN1 for 7 days after the attack of colitis, an amelioration in the clinical manifestations and histological signs became apparent, as assessed by the colon length change, DAI and histological scores, and the level of TNF-α. Data from experiments *in vivo* suggested that TNF-α might have both direct and indirect effects on the pathogenesis of colitis. The IBD pathogenesis was also correlated with increased cytokine production in the colon. The inflammatory process has been ameliorated using the specific antibodies for neutralization of TNF-α, IL-6, and IL-1β^26^,^27^. IFN-γ is predominantly secreted by macrophages, natural killer and T cells, and medications targeting IFN-γ are currently undergoing clinical trials^28^. In our study, treatment with H-SN1 was shown to reduce the levels of IL-1β, IFN-γ, and IL-6 transcripts in the inflamed colon, and also to inhibit the mRNA expression of TNF-α. The transcriptional inhibition of these proinflammatory mediators by blockage of the TNF-α receptor might be responsible for the significant decreased epithelial cell damage observed in the inflamed colon. Consistent with this, mice with *TNFR1* knockout were shown to exhibit only a trifling mucosal injury and a mild inflammatory cell infiltration in response to DSS^29^. Furthermore, IL-10, as an anti-inflammatory cytokine, is secreted by several different cell types including epithelial cells, T cells, macrophages, and dendritic cells. The cure of murine colitis depends upon the production of IL-10; notably, our results demonstrated that treatment with 10 μg/kg H-SN1 could maintain a significantly higher mRNA expression level of IL-10 in the inflamed colon than in the control (Fig. 6G). Therefore, the suppression of the mRNA level of pro-inflammatory mediators and the enhancement of anti-inflammatory cytokine mRNA expression would likely lead to a decrease of inflammation in the colonic mucosa, which might represent the mechanisms by which H-SN1 treatment attenuated DSS-induced colitis in our study.

Because H-SN1 is a TNFR1-binding peptide, we also investigated the effect of H-SN1 on TNF-α-mediated NF-κB and MAPK pathway activation in the colonic tissue of mice with colitis. Genes encoding proinflammatory mediators such as IL-6, TNF-αand IL-12 are transcriptionally activated in response to TNF-α, primarily through NF-κB activation^30^, and NF-κB activation in Fos−/− mice has been shown to result in augmented inflammatory responses^31^. In our study, we found that the phosphorylation of IκB was reduced (Fig. 6E,F, and Supplementary Fig. S2), which inhibited the activation of NF-κB and impaired the production of pro-inflammatory cytokines. The modulation of TNF-α, a key mediator in the inflammatory process in IBD, is interconnected with MAPK pathways. Because of the key role of the inflammatory process in IBD, the modulation of TNF-α is associated with MAPK pathways. In particular, colonic biopsies displayed enhanced JNK and p38 MAPK activation in the inflamed colonic mucosa of patients with IBD, and the inhibition of these factors represents a novel therapeutic strategy for this disorder^32^,^33^. Here, our results demonstrated that the phosphorylation of JNK, p38 and ERK1/2 was inhibited by treatment with H-SN1.

In conclusion, our investigation demonstrates that H-SN1, a TNFR1-binding peptide screened from a *H. cyanocinctus* venom gland T7 phage display library, represents a candidate TNFR1 inhibitor that inhibits the TNF-α/TNFR1 interaction and the biological functions of TNF-α in *in vitro* and *in vivo* models. Our research found that treatment with H-SN1 exhibited a marked therapeutic effect on the clinical symptoms and histological damage of the inflamed colonic mucosa in DSS-induced murine colitis, and also affects the production of inflammatory mediators (i.e., TNF-α, IL-1β, IL-6, IFN-γ, and IL-10) and suppresses inflammatory cell infiltration. Thus, inhibition of the TNF-α-mediated activation of the NF-κB and MAPK pathways might represent important mechanisms by which H-SN1 is able to alleviate colitis in the DSS model. In summary, H-SN1 might serve as a potent candidate for the treatment of TNF-α-associated inflammatory diseases such as IBDs.

## Materials and Methods

### Reagents and cells

H-SN1 was synthesized and purified up to 95% (APeptide Co., Ltd. Shanghai, China) and was freshly dissolved in normal saline. TNF-α, TNFR1, and TNFR2 were from Peprotech (Rocky Hill, NJ, USA). DSS was from MPBio (36–50 kDa; Solon, OH).

HEK293, HT29, and L929 cell lines were obtained from ATCC. The *Hydrophis cyanocinctus* venom gland T7 phage display library was constructed previously according to the OrientExpress cDNA Manual and the T7Select System Manual (Novagen). The titer of the library was 1.56 × 10^6^ pfu/ml. The *Escherichia coli* strain BLT5403 was used as the host strain for the phage library.

### Construction of the T7 phage display library

Total RNA from the *Hydrophis cyanocinctus* venom gland was extracted from fresh venom gland tissues and purified. The T7Select 10-3 OrientExpress cDNA Cloning System, oligo(dT) (Novagen) was chosen to construct the cDNA library. The recombinant vectors were subsequently packaged with T7 Packaging Extracts (Novagen) and propagated in BLT5403. Finally, the titer of the packaged phage library was determined and the amplified library was stored at −80 °C

### Biopanning

The biopanning procedure comprised three rounds of selection and proceeded following the T7Select System Manual (Novagen). Briefly, 1.0 × 10^8^ phage particles were added into a 96-well plate coated with TNFR1, and then incubated for 1 h at 37 °C. The bound phages were eluted and amplified in *E. coli* BLT5403, and the titer of the eluted buffer and the amplified phage particles were determined. Individual colonies were picked randomly after three rounds of selection and DNA sequencing and BLAST were used to analyze the inserted sequences.

### Structure modeling and molecular docking study

The secondary structure of H-SN1 was predicted using The Protein Structure Prediction (PSIPRED) server. QUARK was used to *ab initio* predict the three dimensional structure of H-SN1 and the lowest energy structure was identified by PROSA, ERRAT, and RAMPAGE servers.

A molecular docking experiment was used to predict the interaction of H-SN1 with TNFR1 (Protein Database [PDB] code: 1TNR) and analyze the binding regions. CABS-dock was first used to predict the initial complex structure of TNFR1 with H-SN1; RosettaDock was employed for further flexible docking majorization. PyMOL software was chosen to visualize these structures.

### Characterization of H-SN1 binding properties by SPR

To evaluate the binding properties of H-SN1, affinity and competition tests were performed by BIAcore (Uppsala, Sweden). TNFR1 proteins were coupled to a sensor chip after activation with EDC/NHS, and excess activated carboxyl groups were blocked with ethanolamine for 7 min following TNFR1 immobilization. Various concentrations of H-SN1 were injected separately over the chip surface with a flow rate of 10 μl/min in PBS. HCl-Gly buffer (pH 2.0) was used to regenerate the chip between binding cycles. The binding curves were analyzed using BIAevaluation 3.2 software. To investigate whether H-SN1 could block TNF-α/TNFR1 association, 20 nM TNF-α, diluted with or without several concentrations of H-SN1 (TNF-α/H-SN1 = 1:0.5 or 1:5) was allowed to pass through the coupled TNFR1.

### Cytotoxicity assays

L929 mouse fibrosarcoma cells were seeded and then incubated for 12 h at 37 °C. Serial concentrations of H-SN1 were dissolved in medium containing 20 ng/ml TNF-α and 0.2 μg/ml actinomycin D, and the TNF-α-free groups were treated with serial dilutions of H-SN1 with or without actinomycin D. The MTT assay was used to determine cytoactivity.

### Western blotting

Total cell or tissue lysates were extracted with lysis buffer, resolved using 10% SDS-PAGE gel, and transferred to a nitrocellulose filter membrane (0.2 μm). The antigens were detected by specific antibodies followed by the respective secondary antibodies. GAPDH was simultaneously detected to compare the protein loaded into each lane. The following antibodies were used: mouse monoclonal anti-phospho-IκB, mouse monoclonal anti-IκB, rabbit monoclonal anti-ERK, and rabbit monoclonal anti-phospho-ERK (all from Cell Signaling Technologies, Beverly, MA, USA); mouse polyclonal anti-phospho-p38, rabbit polyclonal anti-p38, rabbit monoclonal anti-phospho-JNK, and mouse monoclonal anti-JNK (all from ThermoFisher Scientific, Rockford, IL, USA); and rabbit monoclonal anti-GAPDH was from Santa Cruz Biotechnology (Dallas, TX, USA).

### Ethics statement

The snake (*Hydrophis cyanocinctus)* was captured in the South China Sea near Guangxi Province (P. R. China). The venom gland was isolated and immediately frozen in liquid nitrogen. BALB/c mice (6-week-old, male) were purchased from Shanghai SLAC Laboratory Animal Co., Ltd. (Shanghai, China). The Animal Care and Use Committee of the Second Military Medical University approved all the animal tests and experimental protocols including venom gland isolation and the mouse study, which were performed in accordance with the Chinese guidelines for the care and use of laboratory animals.

### Induction of DSS colitis

To induce colitis, BABL/c mice were administered a 2.5% DSS solution in drinking water for 7 consecutive days. The DAI, which is the combined scores of weight loss, stool consistency, and bleeding (Table 1) was monitored during the entire experiment as previously described^34^. On the 7th day, mice were sacrificed, and longitudinal strips of colon tissue were harvested, frozen in liquid nitrogen, and stored at −80 °C for real time PCR and western blot analysis. The remainder of the colon was fixed in formalin for histological processing.

### Groups

Mice were randomized into four groups (6 mice per group). The normal (normal) and DSS (control) control groups received normal saline at a dose of 10 ml/kg; groups 3 and 4 were administered H-SN1 at 5 and 10 μg/kg, respectively. All treatments were continued for 7 consecutive days. Drugs were given by intraperitoneal injection once daily and were dissolved in normal saline.

### Histopathology

Formalin-fixed and paraffin-embedded sections of colonic tissues were stained with hematoxylin and eosin. Histological grading was assessed according to previously described criteria (Table 2)^35^,^36^.

### mRNA analysis

TRIzol Reagent (TaKaRa, Otsu, Shiga, Japan) was used for the extraction of total RNA from colonic tissue specimens. Samples of 1 μg mRNA were reverse-transcribed using the PrimeScript™ RT Master Mix Kit (TaKaRa). Real-time PCR was performed using the SYBR^®^ Premix Ex Taq™ kit (TaKaRa) to analyze the expression levels of the tested genes. The primers used were designed based on published sequences 18,37.

### Statistical analysis

For multiple comparisons, the data are presented as the means ± SEM and assessed by one-way Analysis of Variance (ANOVA). GraphPad Prism 5 software for Windows was used (GraphPad Software, San Diego, CA, USA), *p* < 0.05 was considered statistically significant.

## Additional Information

**How to cite this article**: Zheng, Z. *et al.* Screening of an anti-inflammatory peptide from *Hydrophis cyanocinctus* and analysis of its activities and mechanism in DSS-induced acute colitis. *Sci. Rep.* **6**, 25672; doi: 10.1038/srep25672 (2016).

## Supplementary Material

Supplementary Information

## Figures and Tables

**Table 1:** Scores of Disease Activity Index (DAI).

Score	Body weight loss (%)	Stool consistency	Fecal blood
0	none	normal	none
1	1~5		
2	5~10	loose stools	hemoccult+
3	10~20		
4	>20	diarrhea	gross bleeding

**Table 2:** Histological scores.

Score	Severity of inflammation	Extent of inflammation	Crypt damage
0	none	none	none
1	mild	mucosal	basal 1/3
2	moderate	mucosal and submucosal	basal 2/3
3	severe	transmural	crypts lost but surface epithelium present
4			crypts and surface epithelium lost
