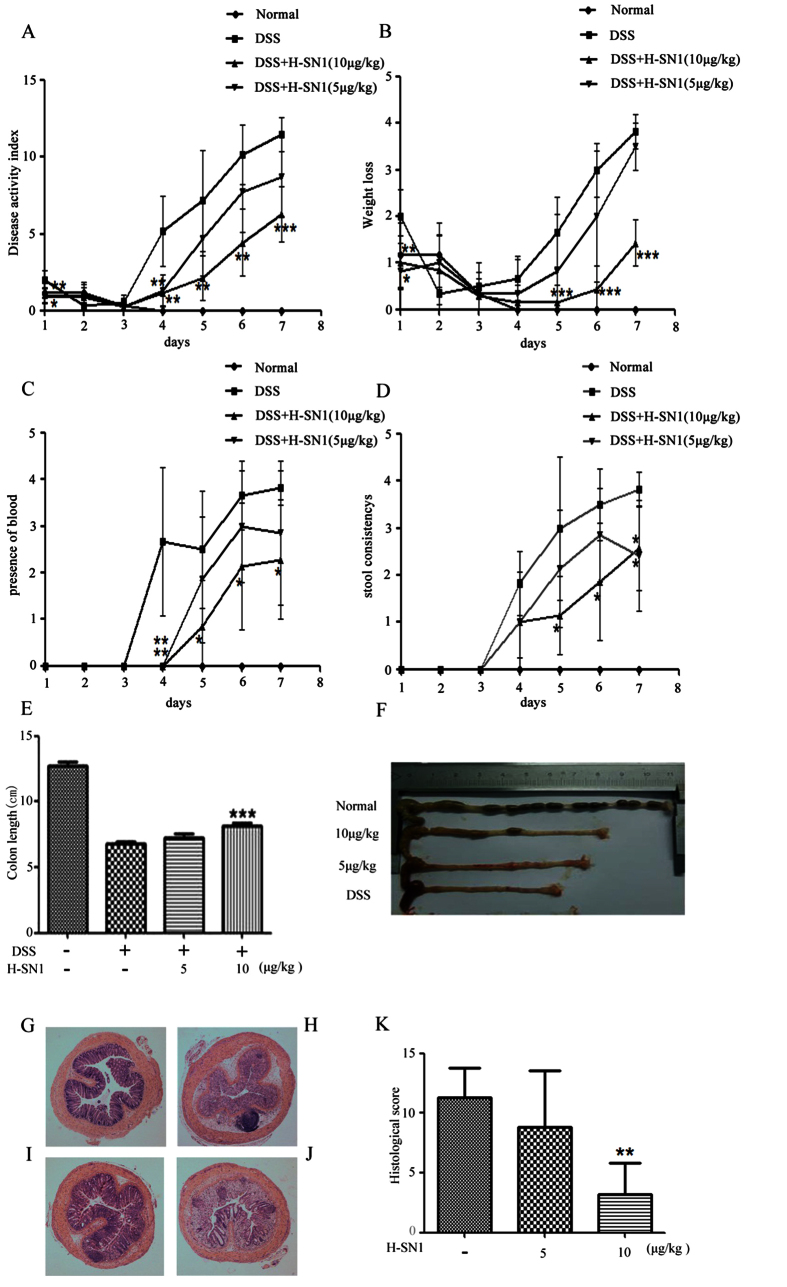# Corrigendum: Screening of an anti-inflammatory peptide from *Hydrophis cyanocinctus* and analysis of its activities and mechanism in DSS-induced acute colitis

**DOI:** 10.1038/srep31259

**Published:** 2016-08-25

**Authors:** Zengjie Zheng, Hailong Jiang, Yan Huang, Jie Wang, Lei Qiu, Zhenlin Hu, Xingyuan Ma, Yiming Lu

Scientific Reports 6: Article number: 25672; 10.1038/srep25672published online: 05 09 2016; updated: 08 25 2016

This Article contains errors.

In the Results section under subheading ‘*H. cyanocinctus* venom gland T7 phage display library construction and biopanning’,

“DEQHLETELHTHLTSVLTANGFQ”

should read:

“DEQHLETELHTLTSVLTANGFQ”

In Figure 5A, 5B, 5C and 5D, the DSS+H-SN1 unit ‘μg/kg’ is incorrectly given as ‘μ/kg’. The correct Figure 5 appears below as Figure 1.

## Figures and Tables

**Figure 1:**