# Supplementary material for: Screening of an anti-inflammatory peptide from Hydrophis cyanocinctus and analysis of its activities and mechanism in DSS-induced acute colitis
Source: Sci Rep. 2016 May 9;6:25672. doi: 10.1038/srep25672 (PMC4860709; doi:10.1038/srep25672)
Supplement: Supplementary Information [file srep25672-s1.pdf]

Screening of an anti-inflammatory peptide from *Hydrophis cyanocinctus* and analysis of its activities and mechanism in DSS-induced acute colitis

Zengjie Zheng[†#], Hailong Jiang [‡#], Yan Huang [‡#], Jie Wang [‡], Lei Qiu [‡], Zhenlin Hu[‡], Xingyuan Ma [†*], Yiming Lu [‡*]

[†] State Key Laboratory of Bioreactor Engineering, East China University of Science and Technology, Shanghai 200237, China

[‡] Department of Biochemical Pharmacy, School of Pharmacy, Second Military Medical University, Shanghai 200433, China

[#] *Contributed equally to this work.*

** Correspondence and requests should be addressed to associate Prof. Yiming Lu and. Prof. Xingyuan Ma; Tel./fax: +862181871333 (Y. LU) and +86 2164250135 (X. MA); E-mail: bluesluyi@sina.com and E-mail: maxy@ecust.edu.cn*

SUPPLEMENTARY INFORMATION includes:

Supplementary Figures S1-S2

A

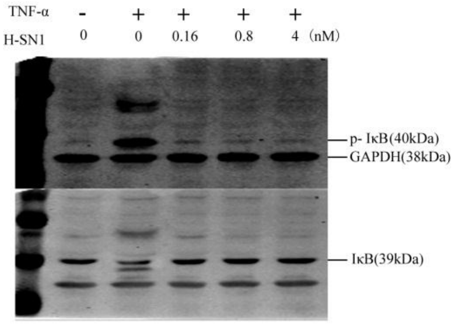

B

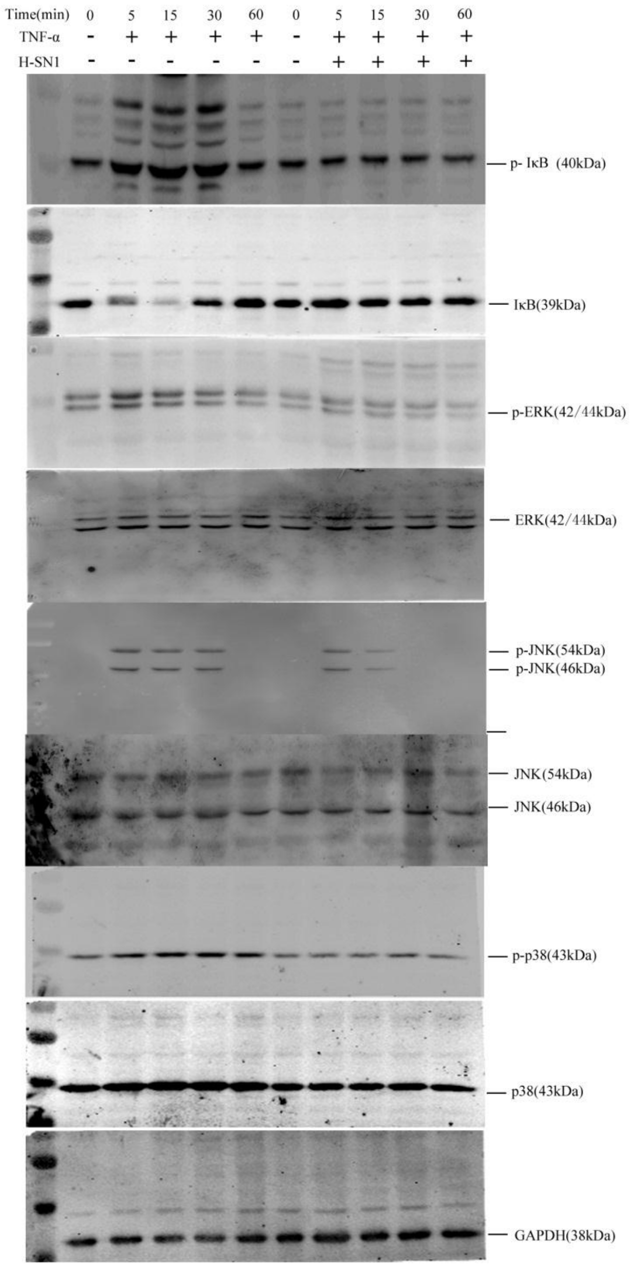
Time(min) 0 5 15 30 60 0 5 15 30 60
TNF-α - + + + + - + + + +
H-SN1 - - - - - - + + + +
p- IκB (40kDa)
IκB(39kDa)
p-ERK(42/44kDa)
ERK(42/44kDa)
p-JNK(54kDa)
p-JNK(46kDa)
JNK(54kDa)
JNK(46kDa)
p-p38(43kDa)
p38(43kDa)
GAPDH(38kDa)

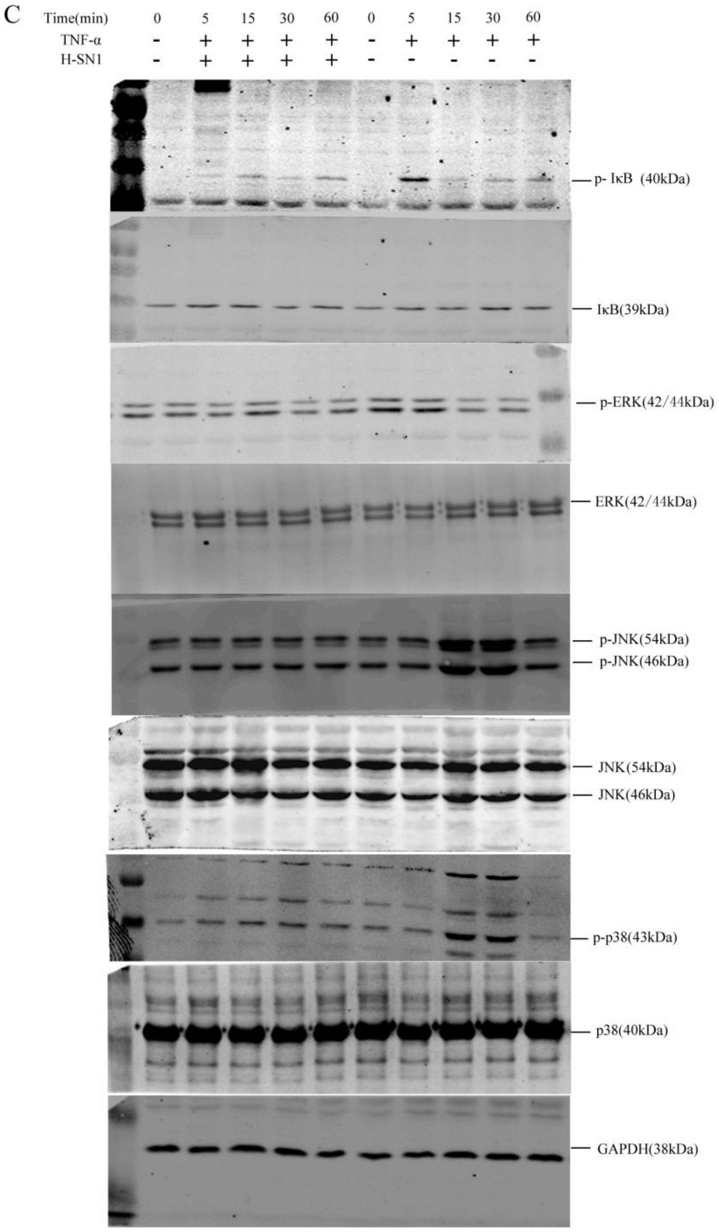

Supplementary Fig. S1:.H-SN1 inhibites TNF-α-induced NF-κB and MAPKs signaling activation. (A) The original blots for Fig. 4A. (B) The original blots for Fig. 4B. (C) The original blots for Fig. 4C.

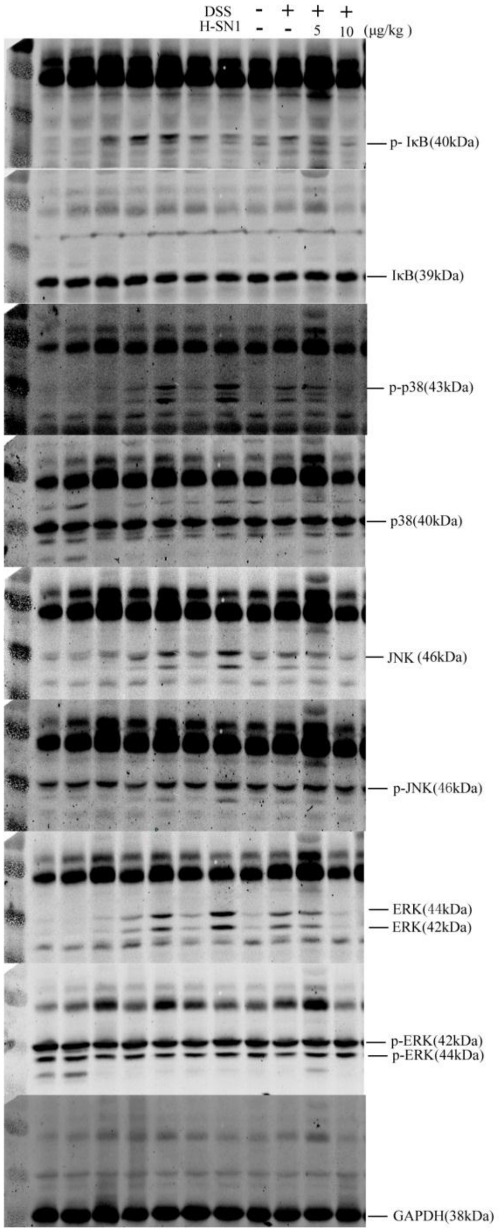
DSS - + + +
H-SN1 - - 5 10 (μg/kg)
p- IκB(40kDa)
IκB(39kDa)
p-p38(43kDa)
p38(40kDa)
JNK (46kDa)
p-JNK(46kDa)
ERK(44kDa)
ERK(42kDa)
p-ERK(42kDa)
p-ERK(44kDa)
GAPDH(38kDa)

Supplementary Fig. S2: The western blot assay of the phospharylation levels of proteins in colon tissues. The original blots for Fig. 6E.
